# Association of high-sensitivity C-reactive protein with hepatic fibrosis in patients with metabolic dysfunction-associated steatotic liver disease

**DOI:** 10.3389/fimmu.2025.1544917

**Published:** 2025-02-10

**Authors:** Yunfei Wu, Guojun Zheng, Fan Zhang, Wenjian Li

**Affiliations:** ^1^ Department of Pathology, Changzhou Third People’s Hospital, Changzhou, China; ^2^ Changzhou Clinical College, Xuzhou Medical University, Changzhou, China; ^3^ Clinical Laboratory, Changzhou Third People’s Hospital, Changzhou, China; ^4^ Department of Endocrinology, Changzhou Third People’s Hospital, Changzhou, China; ^5^ Department of Clinical Nutrition, Changzhou Third People’s Hospital, Changzhou, China; ^6^ Department of Urology, Changzhou Third People’s Hospital, Changzhou, China

**Keywords:** high-sensitivity C-reactive protein, metabolic dysfunction-associated steatotic liver disease, hepatic fibrosis, cross-regional study, dose-response relationship

## Abstract

**Objective:**

This study aimed to investigate the association between high-sensitivity C-reactive protein (hsCRP) levels and hepatic fibrosis in patients with metabolic dysfunction-associated steatotic liver disease (MASLD) and assess its predictive efficacy.

**Methods:**

The study included 1,477 participants from the United States and 1,531 from China diagnosed with MASLD. Liver stiffness measurement (LSM) and controlled attenuation parameter (CAP) were assessed by vibration-controlled transient elastography (VCTE) to evaluate the presence and degree of hepatic fibrosis and steatosis. The relationship between hsCRP levels and hepatic fibrosis in MASLD patients was examined using multivariable-adjusted and restricted cubic spline (RCS) models. Additionally, subgroup analyses were conducted to investigate the potential heterogeneity among different characteristic subgroups.

**Results:**

The results demonstrated a significant correlation between elevated hsCRP levels and an increased risk of significant fibrosis, advanced fibrosis, and cirrhosis in the US cohort of MASLD patients (OR 2.22, 1.69, and 2.85, respectively; all P <0.05). The results of the Chinese cohort were consistent with those of the US cohort, and there was a significant and positive correlation between hsCRP levels and the risk of hepatic fibrosis in patients with MASLD (OR 2.53, 3.85, and 3.78, respectively, all P <0.001). The RCS analysis revealed a significant non-linear relationship between hsCRP levels and the degree of hepatic fibrosis, with disparate inflection point values observed across different cohorts (approximately 9 mg/L in the US cohort and 4 mg/L in the Chinese cohort). The impact of hsCRP levels on the risk of hepatic fibrosis varied across different subgroups with distinct characteristics.

**Conclusion:**

The present study demonstrated a significant correlation between hsCRP levels and the degree of hepatic fibrosis in patients with MASLD, with notable dose-response relationships and subgroup differences.

## 1 Introduction

Metabolic dysfunction-associated steatotic liver disease (MASLD) is a modern lifestyle disease that has emerged as a significant public health concern, with a marked increase in prevalence across the globe in recent years (1–4). MASLD not only disrupts the normal physiological function of the liver but also frequently coexists with components of metabolic syndrome, including obesity, diabetes mellitus (DM), hypertension, and dyslipidemia. These comorbidities further elevate the risk of cardiovascular disease (1, 3, 5–8). In the pathological progression of MASLD, hepatic fibrosis represents a pivotal stage whereby the liver’s repair response to chronic injury, specifically the aberrant proliferation of intrahepatic connective tissue and its gradual replacement of normal liver tissue, becomes evident. In the absence of timely and effective intervention, hepatic fibrosis will continue to deteriorate, potentially leading to the development of cirrhosis or even hepatocellular carcinoma (2, 5, 9, 10). This can have a significantly detrimental impact on the lives and health of patients.

The pathogenesis of hepatic fibrosis is a complex and intricate process involving many factors, such as inflammation, oxidative stress, and abnormalities in lipid metabolism. Among these, inflammation plays a pivotal role at the core of the disease (9, 11, 12). As a sensitive inflammatory marker, the high-sensitivity C-reactive protein (hsCRP) concentration in the blood can be a sensitive indicator of the body’s low-grade inflammatory response (13, 14). A substantial body of evidence indicates that hsCRP levels are strongly linked to the onset of cardiovascular disease, diabetes, and its associated complications (15–17). In recent years, there has been a notable increase in research activity concerning the role of hsCRP in the study of non-alcoholic fatty liver disease (NAFLD) and its complications. Several studies have demonstrated that elevated hsCRP levels are positively correlated with the severity of NAFLD and the progression of hepatic fibrosis, indicating that inflammatory responses play a pivotal role in the pathological process of NAFLD (18–21). Nevertheless, research examining the correlation between hsCRP and hepatic fibrosis in patients with MASLD remains limited. In contrast to NAFLD, MASLD emphasizes the pivotal role of metabolic irregularities in the pathogenesis of the disease, encompassing a spectrum of metabolic abnormalities such as obesity, insulin resistance, dyslipidemia, and other metabolic disorders. Although there are numerous similarities between MASLD and NAFLD regarding the underlying pathophysiological mechanisms, there may be notable differences between the two regarding the clinical manifestations, rate of disease progression, and incidence of complications. It is, therefore, of great significance to explore the association between MASLD and hsCRP to deepen the understanding of MASLD and optimize its prevention and treatment strategies.

To address this research gap, this study examined the relationship between hsCRP levels and hepatic fibrosis in patients with MASLD. To this end, data from two distinct cohorts were integrated: the National Health and Nutrition Examination Survey (NHANES) cohort in the United States and the Third People’s Hospital Cohort in Changzhou, China. The NHANES cohort, as one of the most representative national health surveys in the United States, provides a wealth of cross-sectional data, which can facilitate a comprehensive understanding of the prevalence of MASLD and its associated complications. The Changzhou Third People’s Hospital cohort, on the other hand, provided pertinent data from the Chinese population, enabling this study to transcend geographical boundaries and enhance the representativeness and generalizability of the results.

This study aimed to verify whether hsCRP levels are associated with an increased risk of hepatic fibrosis in patients with MASLD and to elucidate the underlying mechanisms of this association, the dose-response relationship, and the differences in different population subgroups. The central inquiries of this study are as follows: (1) Is there a correlation between hsCRP levels and the degree of hepatic fibrosis in patients with MASLD? (2) Can hsCRP independently predict hepatic fibrosis in patients with MASLD? By constructing a multivariable adjustment model and a restricted cubic spline model, this study aimed to explore the possible independent association and dose-response relationship between the two to provide new insights and rationale for the clinical management of MASLD.

## 2 Materials and methods

### 2.1 Study population

The data for this study were derived from two distinct cohorts: the NHANES cohort from 2017 to 2018 in the United States and the Changzhou Third People’s Hospital cohort in China from 2018 to 2023. The NHANES database contains data from cross-sectional surveys conducted every two years by the Centers for Disease Control and Prevention (CDC). The study protocol for the database was approved by the Ethics Review Committee of the National Center for Health Statistics (NCHS), and all participants provided informed consent. Following NIH regulations, the NHANES data, which were not collected through direct interaction with participants, could be utilized directly for data analysis without further review by the institutional ethics committee. Given the considerations above, the Ethics Committee of Changzhou Third People’s Hospital concluded that no further ethical review was necessary for the NHANES data utilized in this study. Additionally, the study of the Chinese cohort was also approved by the Ethics Committee of Changzhou Third People’s Hospital. This study was conducted by the principles outlined in the Declaration of Helsinki.

**Figure 1:**
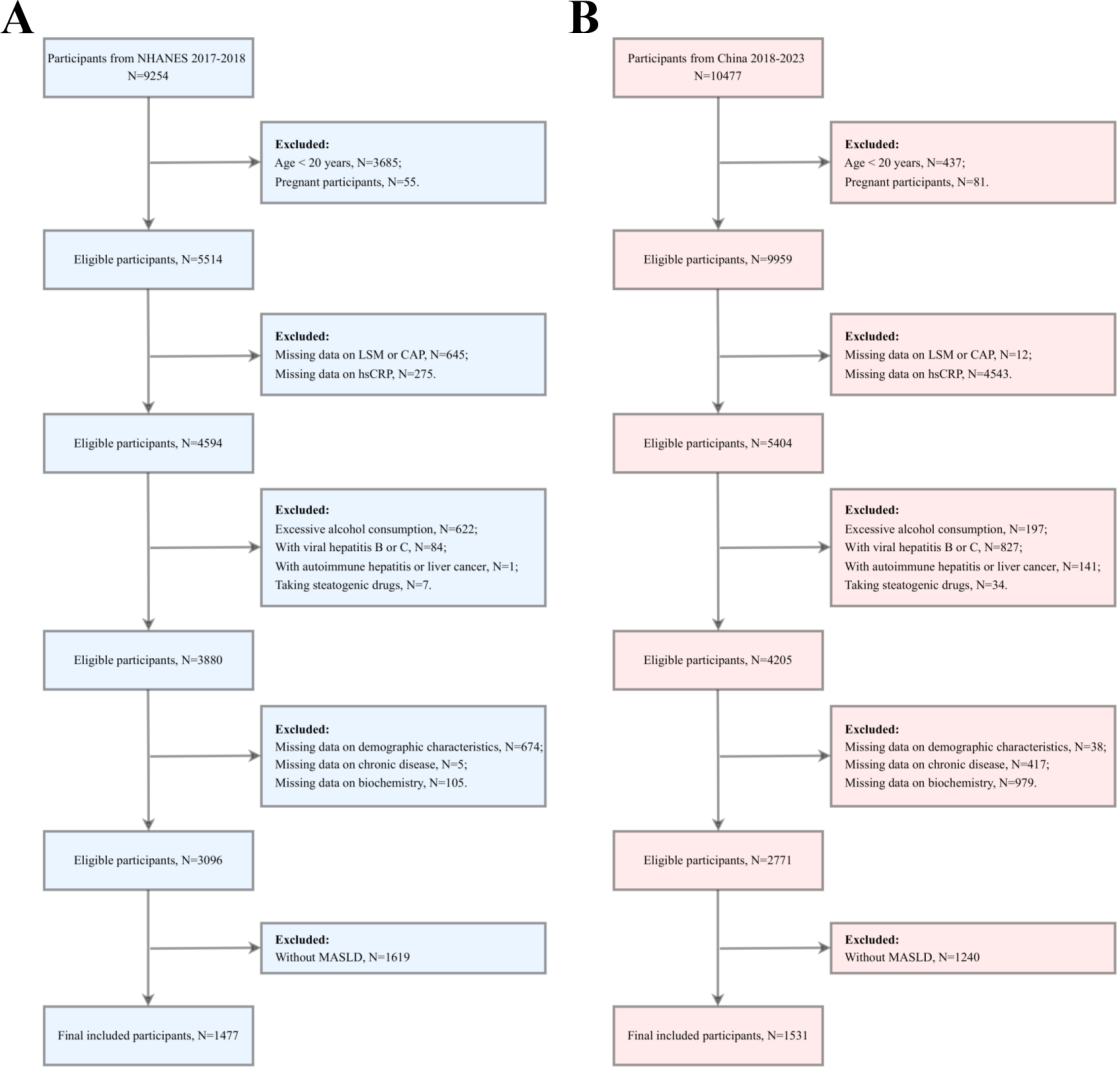
Participant screening flowchart. **(A)** the US cohort; **(B)** the Chinese cohort. LSM, Liver stiffness measurement; CAP, Controlled attenuation parameter; hsCRP, high-sensitivity C-reactive protein; MASLD, Metabolic dysfunction-associated steatotic liver disease.

The study included 9,254 US participants and 10,477 Chinese participants. During the screening process, the following participants were excluded: those under the age of 20 or pregnant, those with no liver stiffness measurement (LSM) or controlled attenuation parameter (CAP) data or no hsCRP data, those with excessive alcohol consumption, and those with viral hepatitis B or C. Additionally, participants with any history of autoimmune hepatitis or hepatocellular carcinoma, those who had taken any medications that may cause fatty liver (e.g., amiodarone, methotrexate, and tamoxifen) within the three months before survey recruitment, and those with missing demographic data, chronic disease data, or critical biochemical markers were excluded. Non-MASLD participants were also excluded. After a comprehensive screening process, 1,477 US and 1,531 Chinese participants were ultimately included in the study for data analysis (**Figure 1**).

### 2.2 Assessment of MASLD and hepatic fibrosis

Vibration-controlled transient elastography (VCTE) was conducted to evaluate the degree of hepatic steatosis, with CAP measurements taken for this purpose. Each participant’s CAP value of 269 dB/m or greater indicated hepatic steatosis (22). Furthermore, a diagnosis of MASLD was confirmed if any of the following five cardiometabolic criteria were met: (1) A body mass index (BMI) of 25 kg/m² or greater or a waist circumference (WC) of 94 cm or greater for males and 80 cm or greater for females; (2) a fasting plasma glucose (FPG) level of 100 mg/dL or greater, or a two-hour post-load blood glucose level of 140 mg/dL or greater, or a glycated hemoglobin (HbA1C) level of 5.7% or greater, or a diagnosis of DM or on glucose-lowering therapy for DM; (3) a blood pressure reading of ≥130/85 mmHg, or the use of antihypertensive medication; (4) a triglyceride (TG) level ≥150 mg/dL or the use of lipid-lowering therapy; (5) a high-density lipoprotein cholesterol (HDL-C) level <40 mg/dL in men or an HDL-C level <50 mg/dL in women or the use of lipid-lowering therapy (5).

Hepatic fibrosis was evaluated based on LSM values. An LSM of ≥7.6 was considered indicative of significant hepatic fibrosis (F2), while an LSM of ≥9.8 was indicative of advanced hepatic fibrosis (F3). An LSM of ≥12.9 was indicative of cirrhosis (F4) (22).

### 2.3 hsCRP assessment

In the United States cohort, the hsCRP assay during the 2017-2018 cycle was conducted using a near-infrared particle immunoassay rate method with a Roche Cobas 6000 chemistry analyzer (Cobas 6000), as detailed in the Laboratory Methods Documentation section of the NHANES. The hsCRP assay for the Chinese cohort was based on an immunoscattering turbidimetric method, with measurements taken using a Lifotronic Specific Protein Analyzer (PA-990Pro).

### 2.4 Assessment of covariates

In this study, the covariates included gender (male/female), age (years), smoking status (yes/no), drinking habits (yes/no), and history of chronic diseases such as DM, hypertension, and dyslipidemia. DM was determined based on the participant’s professional doctor’s diagnosis, FPG level of 126 mg/dl or more, HbA1c level of not less than 6.5%, and treatment with diabetes medication or insulin. Hypertension was identified based on the participant’s self-reported medical history or current prescription for hypertension medication. Dyslipidemia was defined as the presence of one or more of the following in participants: total cholesterol (TC) ≥200 mg/dL, TG ≥150 mg/dL, HDL-C <50 mg/dL (in women) or <40 mg/dL (in men), and low-density lipoprotein cholesterol (LDL-C) ≥130 mg/dL.

### 2.5 Statistical analysis

This study assessed normality for continuous variables using the Kolmogorov-Smirnov test. Variables that exhibited a normal distribution were expressed as mean ± standard deviation, whereas those that did not conform to a normal distribution were described using the median (and 25th to 75th percentile). To compare the differences between these variables, one-way ANOVA or Kruskal-Wallis tests were selected for statistical analysis based on the distributional characteristics of the data. Categorical variables were presented as frequencies and percentages, and the chi-square test was employed to compare differences between groups.

We constructed a logistic regression model to investigate the potential association between hsCRP and hepatic fibrosis in patients with MASLD. We calculated the odds ratio (OR) and its 95% confidence interval (CI). We constructed multivariable-adjusted models to assess this relationship more accurately and control for potential confounding variables. Specifically, Model 1 was the unadjusted base model; Model 2 incorporated gender and age as adjustment variables based on Model 1; and Model 3 further adjusted for smoking, alcohol consumption, diabetes, hypertension, and dyslipidemia based on Model 2. Furthermore, we employed the restricted cubic spline (RCS) model to investigate the potential dose-response relationship between hsCRP and hepatic fibrosis in MASLD patients. Based on the inflection point values obtained from the RCS analysis, the data were divided into two intervals and subjected to further analysis using segmented logistic regression. This allowed for a more detailed examination of the associations between the predictor variables and the results of each segment.

To investigate the relationship between hsCRP and the risk of hepatic fibrosis in MASLD patients across different subgroups, we conducted a subgroup analysis according to gender (male/female), smoking status (yes/no), alcohol consumption (yes/no), presence of DM (yes/no), hypertension (yes/no), and dyslipidemia (yes/no), and performed an interaction analysis. To assess the efficacy of hsCRP in predicting the degree of hepatic fibrosis in patients with MASLD, we employed receiver operating characteristic (ROC) curve analysis.

All statistical analyses employed a two-sided test, and a P-value of less than 0.05 was used as the threshold for determining statistical significance. All statistical analyses were conducted using R 4.4.0 (R Foundation, http://www.R-project.org) and SPSS 23.0 (IBM Corporation, Armonk, NY, USA) software. GraphPad Prism version 9.0 (GraphPad Software, Inc., USA) facilitated the generation of graphical representations.

## 3 Results

### 3.1 Baseline characteristics of patients with MASLD based on hsCRP quartiles in the US cohort

The results demonstrated a statistically significant reduction in the proportion of male patients (from 62.30% to 35.04%, P < 0.001) with increasing hsCRP levels. The median age decreased (57.00 to 52.00 years, P < 0.001). The proportions of alcohol consumption, hypertension, DM, and dyslipidemia differed significantly among the different hsCRP quartiles (P values of 0.004, 0.029, <0.001, and <0.001, respectively). Furthermore, BMI, WC, HbA1c, TC, white blood cells (WBC), neutrophils, lymphocytes, monocytes, platelets, gamma-glutamyltransferase (GGT), LSM, and CAP increased with elevated hsCRP levels (all P <0.05). Conversely, HDL-C levels exhibited a decline. Regarding hepatic fibrosis, the prevalence of significant fibrosis, advanced fibrosis, and cirrhosis demonstrated a notable increase with elevated hsCRP levels (P values of <0.001, 0.005, and <0.001, respectively) (**Table 1**).

**Table 1:** Baseline characteristics of MASLD patients based on hsCRP quartiles in the US cohort.

Variables	hsCRP				
	Quartile 1 (n = 366)	Quartile 2 (n = 372)	Quartile 3 (n = 368)	Quartile 4 (n = 371)	*P*
Gender, n (%)					<0.001
Male	228 (62.30)	222 (59.68)	183 (49.73)	130 (35.04)	
Female	138 (37.70)	150 (40.32)	185 (50.27)	241 (64.96)	
Age (years)	57.00 (43.00,68.00)	56.00 (42.00,68.00)	52.00 (41.00,64.00)	52.00 (39.00,62.00)	<0.001
Smoke, n (%)					0.276
Yes	156 (42.62)	145 (38.98)	155 (42.12)	171 (46.09)	
No	210 (57.38)	227 (61.02)	213 (57.88)	200 (53.91)	
Alcohol, n (%)					0.004
Yes	193 (52.73)	179 (48.12)	173 (47.01)	147 (39.62)	
No	173 (47.27)	193 (51.88)	195 (52.99)	224 (60.38)	
Hypertension, n (%)					0.029
Yes	147 (40.16)	176 (47.31)	186 (50.54)	180 (48.52)	
No	219 (59.84)	196 (52.69)	182 (49.46)	191 (51.48)	
Diabetes, n (%)					<0.001
Yes	89 (24.32)	112 (30.11)	103 (27.99)	150 (40.43)	
No	277 (75.68)	260 (69.89)	265 (72.01)	221 (59.57)	
Dyslipidemia, n (%)					<0.001
Yes	250 (68.31)	277 (74.46)	296 (80.43)	292 (78.71)	
No	116 (31.69)	95 (25.54)	72 (19.57)	79 (21.29)	
BMI (kg/m^2^)	28.60 (26.30,32.10)	30.55 (28.00,34.20)	33.45 (29.10,37.70)	36.90 (31.75,42.80)	<0.001
WC (cm)	99.70 (94.53,108.80)	105.60 (98.55,114.82)	110.55 (101.25,120.90)	117.50 (106.20,128.40)	<0.001
FPG (mg/dL)	96.00 (89.00,106.75)	96.00 (89.00,110.25)	97.00 (89.75,110.00)	99.00 (90.00,118.50)	0.064
HbA1c (%)	5.70 (5.40,6.00)	5.70 (5.40,6.20)	5.80 (5.40,6.20)	5.90 (5.50,6.70)	<0.001
TC (mg/dL)	185.50 (160.00,219.75)	190.00 (161.00,218.00)	195.50 (169.00,221.00)	187.00 (161.00,213.00)	0.036
TG (mg/dL)	133.00 (95.00,196.00)	150.00 (101.00,212.25)	150.00 (106.00,214.25)	139.00 (105.00,189.50)	0.052
HDL-c (mg/dL)	49.00 (41.00,61.00)	46.00 (39.00,55.00)	45.00 (39.00,52.00)	46.00 (38.00,55.00)	<0.001
WBC (10^3^ cells/μL)	6.60 (5.60,8.07)	7.10 (6.10,8.40)	7.50 (6.30,8.90)	8.30 (6.80,10.00)	<0.001
Neutrophils (10^3^ cells/μL)	3.50 (2.90,4.60)	4.10 (3.20,5.10)	4.30 (3.48,5.30)	5.00 (3.80,6.27)	<0.001
Lymphocyte (10^3^ cells/μL)	2.10 (1.70,2.60)	2.20 (1.80,2.70)	2.30 (1.80,2.70)	2.35 (1.80,2.90)	0.001
Monocyte (10^3^ cells/μL)	0.50 (0.40,0.70)	0.60 (0.50,0.70)	0.60 (0.50,0.70)	0.60 (0.50,0.70)	<0.001
Platelet (10^3^ cells/μL)	226.50 (197.00,264.75)	233.00 (193.75,271.00)	242.50 (207.75,281.00)	267.00 (228.00,311.00)	<0.001
AST (U/L)	20.00 (17.00,24.75)	21.00 (17.00,25.00)	20.00 (17.00,26.25)	18.00 (14.00,24.00)	<0.001
ALT (U/L)	21.00 (15.00,29.00)	22.00 (16.00,32.00)	22.00 (16.00,31.25)	19.00 (14.00,29.00)	0.003
GGT (IU/L)	22.50 (17.00,33.00)	26.00 (18.00,41.00)	26.00 (19.00,42.00)	27.00 (19.00,43.00)	<0.001
LSM (kpa)	5.15 (4.20,6.30)	5.40 (4.40,6.80)	5.60 (4.60,7.20)	5.80 (4.80,7.70)	<0.001
CAP (dB/m)	301.00 (283.25,330.00)	309.00 (287.00,338.00)	316.00 (291.00,352.00)	326.00 (299.50,359.00)	<0.001
Significant fibrosis, n (%)					<0.001
Yes	53 (14.48)	66 (17.74)	83 (22.55)	103 (27.76)	
No	313 (85.52)	306 (82.26)	285 (77.45)	268 (72.24)	
Advanced fibrosis, n (%)					0.005
Yes	31 (8.47)	31 (8.33)	54 (14.67)	52 (14.02)	
No	335 (91.53)	341 (91.67)	314 (85.33)	319 (85.98)	
Cirrhosis, n (%)					<0.001
Yes	9 (2.46)	15 (4.03)	32 (8.70)	27 (7.28)	
No	357 (97.54)	357 (95.97)	336 (91.30)	344 (92.72)	

Data are shown as median (25th, 75th percentiles) or percentages, *p <*0.05 considered statistically signiﬁcant.

MASLD, Metabolic dysfunction-associated steatotic liver disease; hsCRP, high-sensitivity C-reactive protein; BMI, Body mass index; WC, Waist circumference; FPG, Fasting plasma-glucose; HbA1c, Hemoglobin A1c; TC, Total cholesterol; TG, Triglyceride; HDL-C, High density lipoprotein cholesterol; WBC, White blood cell; AST, Aspartate aminotransferase; ALT, Alanine transaminase; GGT, Gamma-glutamyl transferase; LSM, Liver stiffness measurement; CAP, Controlled attenuation parameter.

### 3.2 Baseline characteristics of MASLD patients based on hsCRP quartiles in the Chinese cohort

In the Chinese cohort, the results demonstrated no statistically significant difference in the proportion of male and female patients between different hsCRP quartiles (P = 0.071). The median age was similar between the groups (P = 0.667). The proportions of patients who smoke, consume alcohol, hypertension, and dyslipidemia did not differ significantly between hsCRP quartiles (P values of 0.604, 0.407, 0.481, and 0.116, respectively). However, the proportion of DM increased with hsCRP levels (P = 0.019). Additionally, there was a significant positive correlation between hsCRP levels and the following variables: BMI, WC, HbA1c, TG, GGT, and LSM (all P < 0.05). Conversely, there was a significant negative correlation between hsCRP levels and HDL-C levels (P < 0.05). The prevalence of significant fibrosis, advanced fibrosis, and cirrhosis exhibited a notable increase with elevated hsCRP levels (all P < 0.001) (**Table 2**).

**Table 2:** Baseline characteristics of MASLD patients based on hsCRP quartiles in the Chinese cohort.

Variables	hsCRP				
	Quartile 1 (n = 383)	Quartile 2 (n = 381)	Quartile 3 (n = 384)	Quartile 4 (n = 383)	*P*
Gender, n (%)					0.071
Male	231 (60.31)	217 (56.96)	254 (66.15)	231 (60.31)	
Female	152 (39.69)	164 (43.04)	130 (33.85)	152 (39.69)	
Age (years)	42.00 (32.00,53.00)	44.00 (33.00,53.00)	41.00 (33.00,54.00)	42.00 (34.00,53.00)	0.667
Smoke, n (%)					0.604
Yes	128 (33.42)	126 (33.07)	136 (35.42)	118 (30.81)	
No	255 (66.58)	255 (66.93)	248 (64.58)	265 (69.19)	
Alcohol, n (%)					0.407
Yes	93 (24.28)	75 (19.69)	87 (22.66)	92 (24.02)	
No	290 (75.72)	306 (80.31)	297 (77.34)	291 (75.98)	
Hypertension, n (%)					0.481
Yes	102 (26.63)	99 (25.98)	117 (30.47)	111 (28.98)	
No	281 (73.37)	282 (74.02)	267 (69.53)	272 (71.02)	
Diabetes, n (%)					0.019
Yes	86 (22.45)	98 (25.72)	119 (30.99)	118 (30.81)	
No	297 (77.55)	283 (74.28)	265 (69.01)	265 (69.19)	
Dyslipidemia, n (%)					0.116
Yes	292 (76.24)	304 (79.79)	307 (79.95)	319 (83.29)	
No	91 (23.76)	77 (20.21)	77 (20.05)	64 (16.71)	
BMI (kg/m^2^)	27.20 (24.90,29.70)	27.50 (25.50,30.00)	27.70 (25.58,30.20)	27.70 (25.70,30.60)	0.014
WC (cm)	94.90 (89.65,101.95)	95.50 (89.10,102.50)	96.70 (90.70,105.75)	96.80 (90.25,103.15)	0.008
FPG (mg/dL)	97.20 (90.00,109.80)	97.20 (90.00,113.40)	97.20 (90.00,112.05)	99.00 (90.00,116.37)	0.247
HbA1c (%)	5.88 (5.60,6.35)	5.94 (5.62,6.40)	5.98 (5.66,6.56)	6.02 (5.70,6.57)	0.010
TC (mg/dL)	177.54 (155.69,201.52)	180.64 (155.88,207.71)	180.25 (155.49,206.26)	184.12 (162.26,207.71)	0.199
TG (mg/dL)	138.22 (91.26,202.01)	141.76 (92.14,191.38)	145.30 (105.21,221.72)	168.34 (116.95,241.88)	<0.001
HDL-C (mg/dL)	42.13 (36.72,48.70)	42.13 (35.94,53.72)	41.16 (34.30,49.09)	40.58 (35.17,49.28)	0.023
WBC (10^3^ cells/μL)	6.26 (5.38,7.20)	6.29 (5.31,7.27)	6.19 (5.30,7.20)	6.41 (5.51,7.34)	0.724
Neutrophils (10^3^ cells/μL)	3.55 (3.02,4.26)	3.61 (2.86,4.30)	3.50 (2.83,4.16)	3.66 (2.90,4.21)	0.538
Lymphocyte (10^3^ cells/μL)	2.08 (1.69,2.46)	2.02 (1.66,2.35)	2.05 (1.73,2.46)	2.04 (1.71,2.47)	0.367
Monocyte (10^3^ cells/μL)	0.44 (0.36,0.53)	0.44 (0.37,0.53)	0.43 (0.36,0.51)	0.44 (0.37,0.53)	0.669
Platelet (10^3^ cells/μL)	224.00 (193.00,256.38)	216.00 (180.00,253.00)	221.00 (187.53,261.00)	225.00 (184.00,262.93)	0.277
AST (U/L)	25.00 (19.00,37.00)	26.00 (19.00,40.00)	25.00 (19.00,37.25)	25.00 (18.00,37.50)	0.926
ALT (U/L)	39.00 (22.55,63.99)	36.00 (21.00,63.87)	38.00 (22.00,66.17)	36.80 (23.00,65.50)	0.865
GGT (IU/L)	38.00 (22.40,59.40)	35.90 (23.00,69.50)	41.65 (25.15,75.20)	40.30 (25.00,72.95)	0.043
LSM (kpa)	6.00 (5.00,7.00)	6.30 (5.10,8.40)	6.70 (5.20,9.20)	6.50 (5.20,9.30)	<0.001
CAP (dB/m)	323.00 (298.00,345.00)	324.00 (301.00,352.00)	330.50 (303.00,355.25)	329.00 (300.50,355.00)	0.108
Significant fibrosis, n (%)					<0.001
Yes	68 (17.75)	132 (34.65)	157 (40.89)	135 (35.25)	
No	315 (82.25)	249 (65.35)	227 (59.11)	248 (64.75)	
Advanced fibrosis, n (%)					<0.001
Yes	28 (7.31)	63 (16.54)	78 (20.31)	90 (23.50)	
No	355 (92.69)	318 (83.46)	306 (79.69)	293 (76.50)	
Cirrhosis, n (%)					<0.001
Yes	13 (3.39)	27 (7.09)	26 (6.77)	46 (12.01)	
No	370 (96.61)	354 (92.91)	358 (93.23)	337 (87.99)	

Data are shown as median (25th, 75th percentiles) or percentages, *p <*0.05 considered statistically signiﬁcant.

MASLD, Metabolic dysfunction-associated steatotic liver disease; hsCRP, high-sensitivity C-reactive protein; BMI, Body mass index; WC, Waist circumference; FPG, Fasting plasma-glucose; HbA1c, Hemoglobin A1c; TC, Total cholesterol; TG, Triglyceride; HDL-C, High density lipoprotein cholesterol; WBC, White blood cell; AST, Aspartate aminotransferase; ALT, Alanine transaminase; GGT, Gamma-glutamyl transferase; LSM, Liver stiffness measurement; CAP, Controlled attenuation parameter.

### 3.3 Association of hsCRP with hepatic fibrosis in MASLD patients in the US cohort

The relationship between hsCRP and hepatic fibrosis in patients with MASLD was investigated using multivariable model logistic regression in the US cohort. The results demonstrated that patients in the highest quartile of hsCRP exhibited a markedly elevated risk of significant fibrosis (OR = 2.27, P < 0.001), advanced fibrosis (OR = 1.76, P = 0.018), and cirrhosis (OR = 3.11, P = 0.004) in comparison to those in the lowest quartile of hsCRP. These associations remained significant after adjustment for potential confounding factors, including gender, age, smoking, alcohol consumption, DM, hypertension, and dyslipidemia (OR = 2.22, P < 0.001; OR = 1.69, P = 0.041; OR = 2.85, P = 0.011). These findings suggest that hsCRP may be a valuable predictor of hepatic fibrosis progression in patients with MASLD (**Table 3**).

**Table 3:** Relationship between hsCRP and hepatic fibrosis in patients with MASLD in the US cohort.

Variables	Model 1		Model 2		Model 3	
	OR (95%CI)	*P*	OR (95%CI)	*P*	OR (95%CI)	*P*
Significant fibrosis						
hsCRP						
Quartile 1	1.00 (Reference)		1.00 (Reference)		1.00 (Reference)	
Quartile 2	1.27 (0.86 ~ 1.89)	0.229	1.30 (0.87 ~ 1.93)	0.198	1.20 (0.80 ~ 1.79)	0.374
Quartile 3	1.72 (1.18 ~ 2.52)	0.005	1.90 (1.29 ~ 2.80)	0.001	1.73 (1.17 ~ 2.56)	0.006
Quartile 4	2.27 (1.57 ~ 3.28)	<0.001	2.73 (1.86 ~ 4.01)	<0.001	2.22 (1.49 ~ 3.29)	<0.001
Advanced fibrosis						
hsCRP						
Quartile 1	1.00 (Reference)		1.00 (Reference)		1.00 (Reference)	
Quartile 2	0.98 (0.58 ~ 1.65)	0.947	1.00 (0.59 ~ 1.69)	0.995	0.91 (0.54 ~ 1.54)	0.720
Quartile 3	1.86 (1.16 ~ 2.97)	0.009	2.08 (1.30 ~ 3.35)	0.002	1.84 (1.14 ~ 2.98)	0.013
Quartile 4	1.76 (1.10 ~ 2.82)	0.018	2.17 (1.34 ~ 3.53)	0.002	1.69 (1.02 ~ 2.79)	0.041
Cirrhosis						
hsCRP						
Quartile 1	1.00 (Reference)		1.00 (Reference)		1.00 (Reference)	
Quartile 2	1.67 (0.72 ~ 3.86)	0.233	1.70 (0.73 ~ 3.95)	0.214	1.48 (0.63 ~ 3.45)	0.366
Quartile 3	3.78 (1.78 ~ 8.03)	<0.001	4.24 (1.98 ~ 9.06)	<0.001	3.64 (1.69 ~ 7.85)	<0.001
Quartile 4	3.11 (1.44 ~ 6.72)	0.004	3.86 (1.76 ~ 8.44)	<0.001	2.85 (1.28 ~ 6.37)	0.011

Model 1: crude.

Model 2: adjusted for Gender and Age.

Model 3: adjusted for Gender, Age, Smoke, Alcohol, Diabetes, Hypertension, and Dyslipidemia.

hsCRP, high-sensitivity C-reactive protein; MASLD, Metabolic dysfunction-associated steatotic liver disease; OR, Odds ratio; CI, Confidence interval.

### 3.4 Association of hsCRP with hepatic fibrosis in MASLD patients in the Chinese cohort

A similar investigation was conducted into the relationship between hsCRP levels and hepatic fibrosis in the Chinese cohort. The findings indicated that hsCRP levels were significantly correlated with the risk of significant fibrosis, advanced fibrosis, and cirrhosis after adjustment for various factors. Patients in the higher quartiles exhibited progressively elevated risks of significant fibrosis (OR = 2.53, P < 0.001), advanced fibrosis (OR = 3.85, P < 0.001), and cirrhosis (OR = 3.78, P < 0.001) relative to those in the lowest hsCRP quartile. These results are consistent with those observed in the US cohort, providing further support for the potential of hsCRP as a predictor of hepatic fibrosis in patients with MASLD (**Table 4**).

**Table 4:** Relationship between hsCRP and hepatic fibrosis in patients with MASLD in the Chinese cohort.

Variables	Model 1		Model 2		Model 3	
	OR (95%CI)	*P*	OR (95%CI)	*P*	OR (95%CI)	*P*
Significant fibrosis						
hsCRP						
Quartile 1	1.00 (Reference)		1.00 (Reference)		1.00 (Reference)	
Quartile 2	2.46 (1.75 ~ 3.44)	<0.001	2.45 (1.75 ~ 3.42)	<0.001	2.48 (1.77 ~ 3.49)	<0.001
Quartile 3	3.20 (2.30 ~ 4.46)	<0.001	3.22 (2.31 ~ 4.49)	<0.001	3.22 (2.31 ~ 4.51)	<0.001
Quartile 4	2.52 (1.80 ~ 3.53)	<0.001	2.52 (1.80 ~ 3.52)	<0.001	2.53 (1.80 ~ 3.55)	<0.001
Advanced fibrosis						
hsCRP						
Quartile 1	1.00 (Reference)		1.00 (Reference)		1.00 (Reference)	
Quartile 2	2.51 (1.57 ~ 4.02)	<0.001	2.49 (1.56 ~ 3.99)	<0.001	2.49 (1.55 ~ 3.99)	<0.001
Quartile 3	3.23 (2.04 ~ 5.11)	<0.001	3.26 (2.06 ~ 5.16)	<0.001	3.19 (2.01 ~ 5.07)	<0.001
Quartile 4	3.89 (2.48 ~ 6.12)	<0.001	3.87 (2.46 ~ 6.09)	<0.001	3.85 (2.44 ~ 6.07)	<0.001
Cirrhosis						
hsCRP						
Quartile 1	1.00 (Reference)		1.00 (Reference)		1.00 (Reference)	
Quartile 2	2.17 (1.10 ~ 4.27)	0.025	2.17 (1.10 ~ 4.29)	0.026	2.16 (1.09 ~ 4.28)	0.028
Quartile 3	2.07 (1.05 ~ 4.09)	0.037	2.06 (1.04 ~ 4.10)	0.039	1.97 (0.99 ~ 3.93)	0.055
Quartile 4	3.88 (2.06 ~ 7.32)	<0.001	3.85 (2.03 ~ 7.29)	<0.001	3.78 (1.99 ~ 7.19)	<0.001

Model 1: crude.

Model 2: adjusted for Gender and Age.

Model 3: adjusted for Gender, Age, Smoke, Alcohol, Diabetes, Hypertension, and Dyslipidemia.

hsCRP, high-sensitivity C-reactive protein; MASLD, Metabolic dysfunction-associated steatotic liver disease; OR, Odds ratio; CI, Confidence interval.

### 3.5 RCS analysis

**Figure 2:**
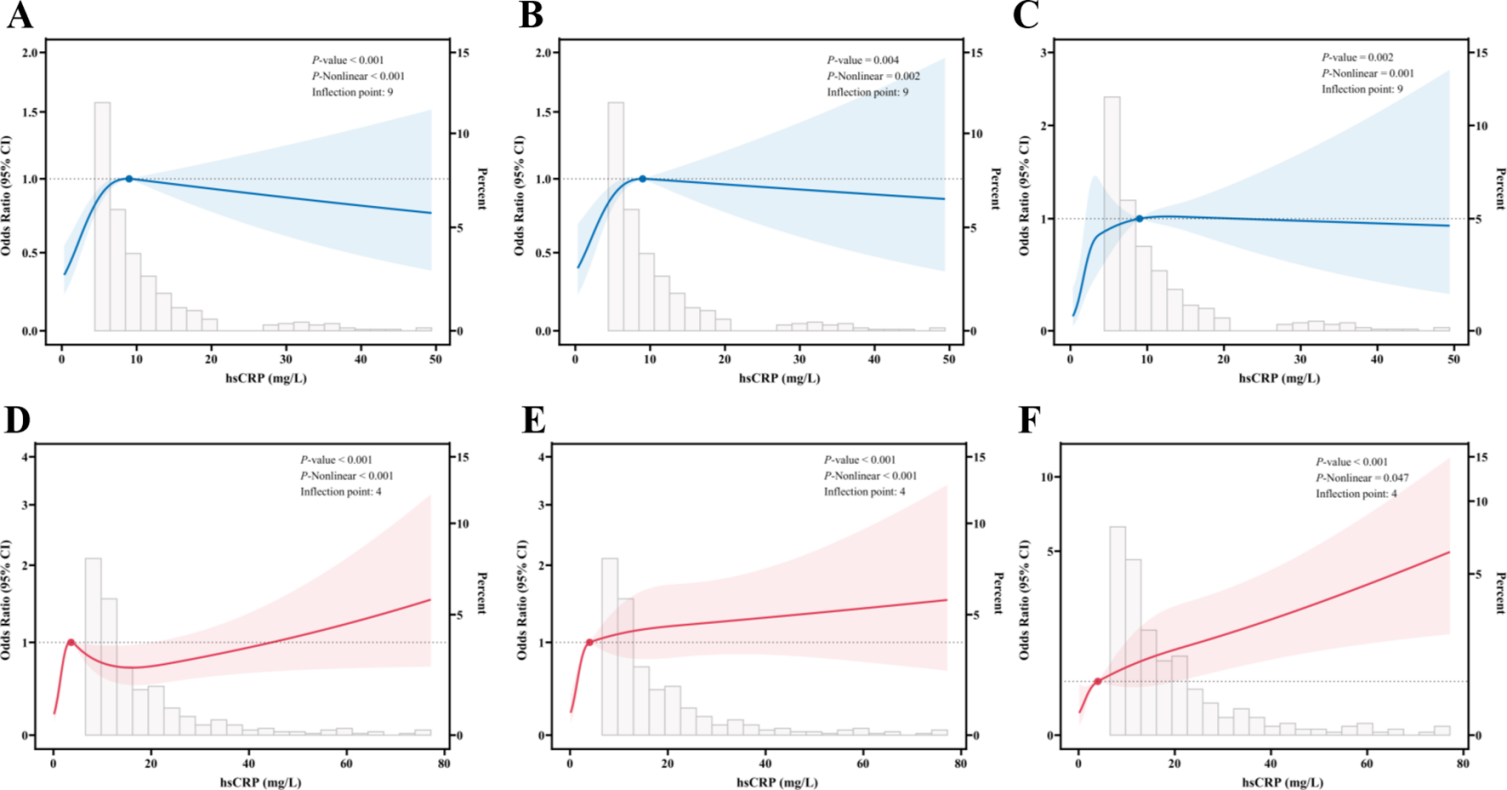
Restricted cubic spline fitting for the association between hsCRP and hepatic fibrosis in patients with MASLD. **(A)** association between hsCRP and significant fibrosis in the US cohort; **(B)** association between hsCRP and advanced fibrosis in the US cohort; **(C)** association between hsCRP and cirrhosis in the US cohort; **(D)** association between hsCRP and significant fibrosis in the Chinese cohort; **(E)** association between hsCRP and advanced fibrosis in the Chinese cohort; **(F)** association between hsCRP and cirrhosis in the Chinese cohort. The solid line displays the odds ratio, with the 95% CI represented by shading. They were adjusted for gender, age, smoke, alcohol, diabetes, hypertension, and dyslipidemia. hsCRP, high-sensitivity C-reactive protein; MASLD, Metabolic dysfunction-associated steatotic liver disease; CI, Confidence interval.

In the context of RCS analyses, an investigation was conducted to elucidate the correlation between serum hsCRP levels and the degree of hepatic fibrosis in patients diagnosed with MASLD. All analyses were adjusted for potential confounding factors, including gender, age, smoking status, alcohol consumption, diabetes, hypertension, and dyslipidemia. In the US cohort, significant nonlinear associations were identified between hsCRP levels and significant fibrosis (**Figure 2A**), advanced fibrosis (**Figure 2B**), and cirrhosis (**Figure 2C**) (P < 0.001, P-Nonlinear < 0.001; P = 0.004, P-Nonlinear = 0.002; P = 0.002, P-Nonlinear = 0.001). For significant fibrosis, advanced fibrosis, and cirrhosis, the inflection point occurred at an hsCRP level of approximately 9 mg/L. At levels of hsCRP below 9 mg/L, the OR values for each type of hepatic fibrosis increased significantly with increasing hsCRP levels. When hsCRP levels reached or exceeded 9 mg/L, the OR values tended to flatness or decrease.

In the Chinese cohort, significant nonlinear relationships were also observed between hsCRP levels and significant fibrosis (**Figure 2D**), advanced fibrosis (**Figure 2E**), and cirrhosis (**Figure 2F**) (P < 0.001, P-Nonlinear < 0.001; P < 0.001, P-Nonlinear < 0.001; P < 0.001, P-Nonlinear = 0.047). The inflection point was observed at hsCRP levels of 4 mg/L. At levels of hsCRP less than 4 mg/L, the ORs for each type of hepatic fibrosis increased significantly with increasing hsCRP levels. At hsCRP levels of 4 mg/L or above, the upward trajectory of the ORs for advanced fibrosis and cirrhosis leveled off, while the ORs for significant fibrosis began to decline.

### 3.6 Segmented logistic regression analysis of the effect of hsCRP levels on hepatic fibrosis in patients with MASLD

A segmented logistic regression analysis was conducted to investigate the effect of hsCRP levels on hepatic fibrosis based on the inflection point values identified through RCS analysis. In the US cohort, the results demonstrated that the risk of significant fibrosis (OR = 1.10, P = 0.005), advanced fibrosis (OR = 1.10, P = 0.037), and cirrhosis (OR = 1.12, P = 0.042) was markedly elevated when hsCRP levels <9 mg/L were adjusted for relevant confounding variables. However, these associations were no longer significant when hsCRP levels were ≥9 mg/L (P values of 0.310, 0.960, and 0.730, respectively) (**Table 5**). This indicates the potential existence of a threshold effect of hsCRP levels on hepatic fibrosis.

**Table 5:** Segmented logistic regression analysis of the effect of hsCRP level on hepatic fibrosis in the US cohort.

Variables	OR (95% CI)	*P*	OR per SD (95%CI)	*P*
Significant fibrosis				
hsCRP (< 9 mg/L)	1.10 (1.03 ~ 1.17)	0.005	1.21 (1.06 ~ 1.38)	0.005
hsCRP (≥ 9 mg/L)	0.99 (0.97 ~ 1.01)	0.310	0.83 (0.59 ~ 1.18)	0.310
Advanced fibrosis				
hsCRP (< 9 mg/L)	1.10 (1.01 ~ 1.21)	0.037	1.20 (1.01 ~ 1.43)	0.037
hsCRP (≥ 9 mg/L)	0.99 (0.98 ~ 1.02)	0.960	0.99 (0.69 ~ 1.42)	0.960
Cirrhosis				
hsCRP (< 9 mg/L)	1.12 (1.00 ~ 1.25)	0.042	1.27 (1.01 ~ 1.60)	0.042
hsCRP (≥ 9 mg/L)	0.99 (0.96 ~ 1.03)	0.730	0.91 (0.55 ~ 1.53)	0.730

ORs were adjusted for Gender, Age, Smoke, Alcohol, Diabetes, Hypertension, and Dyslipidemia.

hsCRP, high-sensitivity C-reactive protein; OR, Odds ratio; SD, Standardized; CI, Confidence interval.

In the Chinese cohort, the results demonstrated that after adjusting for potential confounding variables, patients with hsCRP levels below 4 mg/L exhibited a notable increase in the risk of significant fibrosis (OR = 1.41, P< 0.001), advanced fibrosis (OR = 1.32, P = 0.002), and cirrhosis (OR = 1.15, P = 0.037) as hsCRP levels increased. However, when hsCRP levels reached or exceeded 4 mg/L, the increased risk of significant fibrosis and advanced fibrosis was no longer significant (P values of 0.440 and 0.660, respectively), although the risk of cirrhosis remained increased (OR = 1.02, P = 0.016) (**Table 6**). These results further confirm the potential for differences in the predictive value of hsCRP levels at varying thresholds for hepatic fibrosis in patients with MASLD.

**Table 6:** Segmented logistic regression analysis of the effect of hsCRP level on hepatic fibrosis in the Chinese cohort.

Variables	OR (95% CI)	P	OR per SD (95%CI)	P
Significant fibrosis				
hsCRP (< 4 mg/L)	1.41 (1.23 ~ 1.62)	<0.001	1.44 (1.25 ~ 1.66)	<0.001
hsCRP (≥ 4 mg/L)	1.00 (0.99 ~ 1.01)	0.440	1.07 (0.91 ~ 1.26)	0.440
Advanced fibrosis				
hsCRP (< 4 mg/L)	1.32 (1.11 ~ 1.58)	0.002	1.34 (1.11 ~ 1.62)	0.002
hsCRP (≥ 4 mg/L)	1.00 (0.99 ~ 1.01)	0.660	1.04 (0.87 ~ 1.25)	0.660
Cirrhosis				
hsCRP (< 4 mg/L)	1.15 (1.01 ~ 1.32)	0.037	1.28 (1.02 ~ 1.61)	0.037
hsCRP (≥ 4 mg/L)	1.02 (1.00 ~ 1.03)	0.016	1.35 (1.06 ~ 1.73)	0.016

ORs were adjusted for Gender, Age, Smoke, Alcohol, Diabetes, Hypertension, and Dyslipidemia.

hsCRP, high-sensitivity C-reactive protein; OR, Odds ratio; SD, Standardized; CI, Confidence interval.

### 3.7 Subgroup analysis

**Figure 3:**
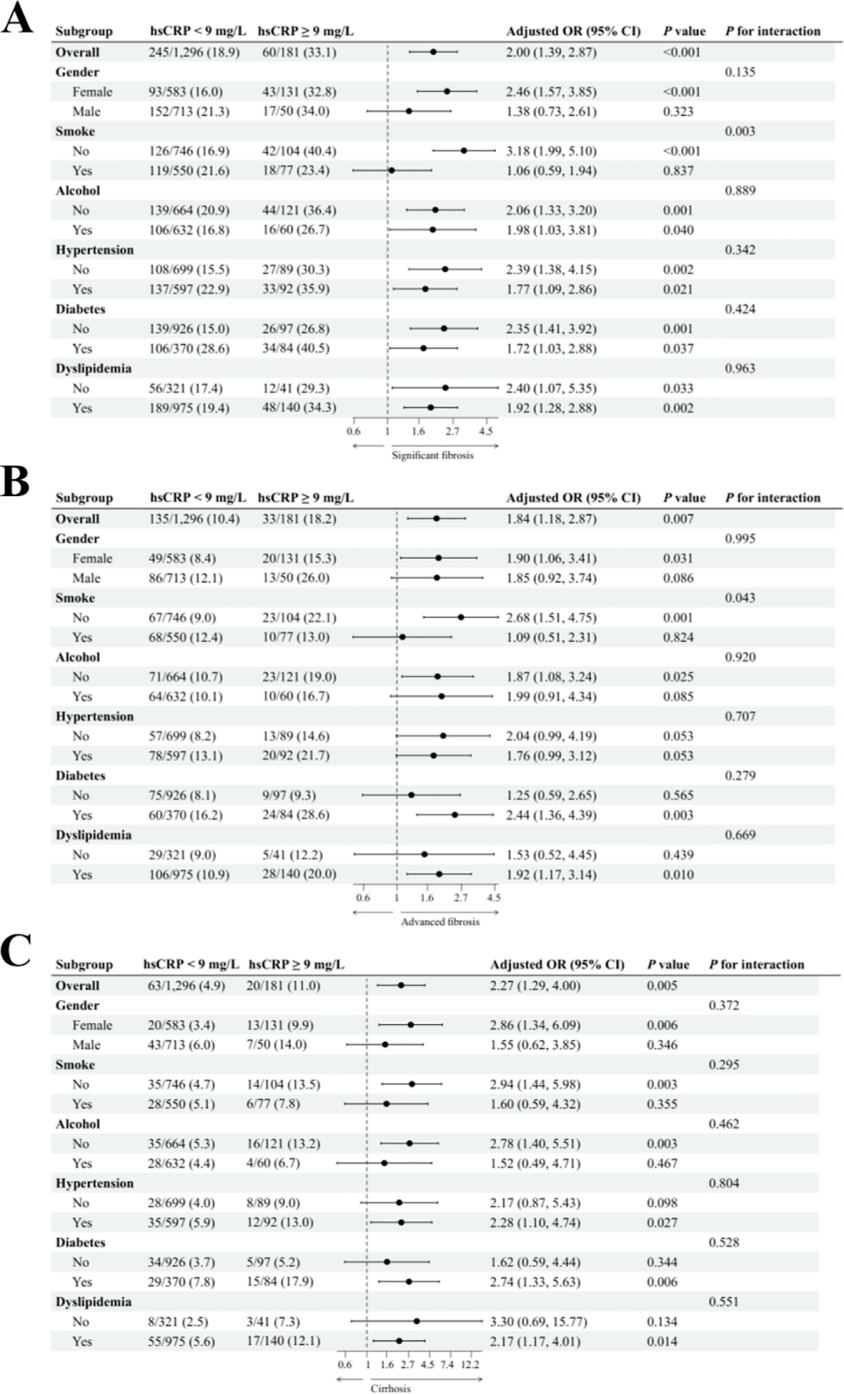
Subgroup analysis of the association between hsCRP and hepatic fibrosis in patients with MASLD in the US cohort. **(A)** association between hsCRP and significant fibrosis; **(B)** association between hsCRP and advanced fibrosis; **(C)** association between hsCRP and cirrhosis. Adjusted variables: gender, age, smoke, alcohol, diabetes, hypertension, and dyslipidemia. The model was not adjusted for the stratification variables themselves in the corresponding stratification analysis. hsCRP, high-sensitivity C-reactive protein; MASLD, Metabolic dysfunction-associated steatotic liver disease; OR, odds ratio; CI, confidence interval.

In the US cohort, we conducted a subgroup analysis to investigate the relationship between hsCRP levels and hepatic fibrosis in patients with MASLD. We adjusted for potential confounding variables, including gender, age, smoking status, alcohol consumption, diabetes, hypertension, and dyslipidemia. The results are presented in **Figure 3**. For significant fibrosis (**Figure 3A**), a significant association was observed between hsCRP levels and the risk of fibrosis. In general, patients with hsCRP levels of 9 mg/L or greater exhibited an adjusted OR of 2.00 (95% CI: 1.39, 2.87) with a P-value of less than 0.001 compared to patients with hsCRP levels below 9 mg/L. In all but the male and smokers subgroups, there was a significant association between hsCRP levels and the risk of fibrosis (P < 0.05). Furthermore, no significant interaction was observed in any subgroup, except for the smokers subgroup. In the analysis of advanced fibrosis (**Figure 3B**), patients with hsCRP levels ≥9 mg/L exhibited an adjusted OR of 1.84 (95% CI: 1.18, 2.87) in comparison to patients with hsCRP levels <9 mg/L, with a P-value of 0.007. In the subgroups of females, non-smokers, non-drinkers, diabetic patients, and patients with dyslipidemia, a significant association was observed between hsCRP levels and the risk of fibrosis (P < 0.05). Nevertheless, no significant interaction was observed in any subgroups except the smoking subgroup. The results of the analyses also supported a significant association between hsCRP levels and the risk of cirrhosis (**Figure 3C**). The adjusted OR was 2.27 (95% CI: 1.29, 4.00), with a P-value of 0.005. The correlation between hsCRP levels and the likelihood of cirrhosis was statistically significant in the following subgroups: females, non-smokers, non-drinkers, and patients with diabetics, hypertension, and dyslipidemia (P < 0.05). No significant interaction was identified in any of the subgroups.

**Figure 4:**
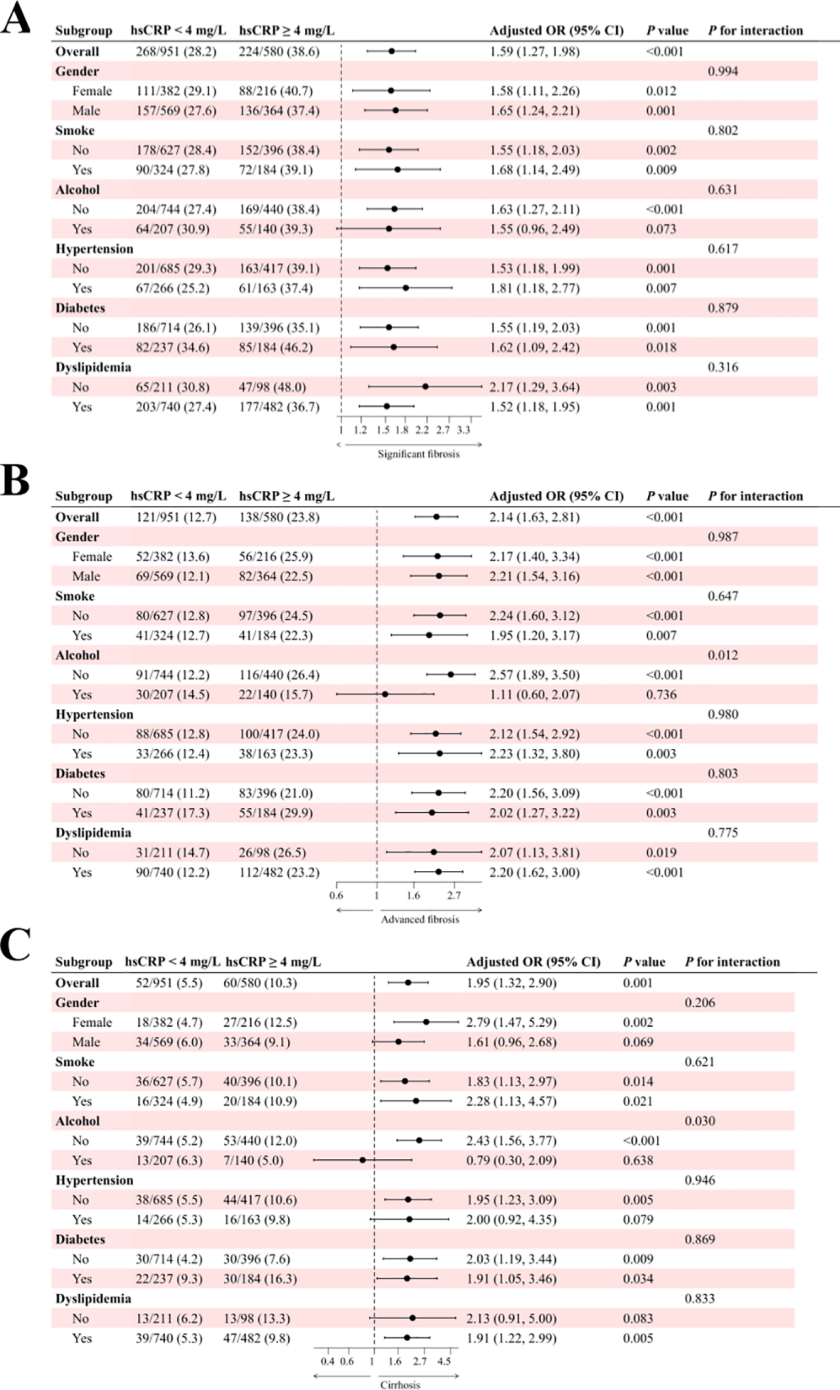
Subgroup analysis of the association between hsCRP and hepatic fibrosis in patients with MASLD in the Chinese cohort. **(A)** association between hsCRP and significant fibrosis; **(B)** association between hsCRP and advanced fibrosis; **(C)** association between hsCRP and cirrhosis. Adjusted variables: gender, age, smoke, alcohol, diabetes, hypertension, and dyslipidemia. The model was not adjusted for the stratification variables themselves in the corresponding stratification analysis. hsCRP, high-sensitivity C-reactive protein; MASLD, Metabolic dysfunction-associated steatotic liver disease; OR, odds ratio; CI, confidence interval.

The results of the subgroup analysis for the Chinese cohort are presented in **Figure 4**. In the study of significant fibrosis (**Figure 4A**), patients with hsCRP levels of 4 mg/L or greater exhibited an adjusted OR of 1.59 (95% CI: 1.27, 1.98) in comparison to patients with hsCRP levels below 4 mg/L, with a P-value of less than 0.001. In all but the drinkers subgroup, there was a significant association between hsCRP levels and the risk of fibrosis (P < 0.05). Furthermore, no significant interaction was observed in any subgroup (P for interaction > 0.05). About advanced fibrosis (**Figure 4B**), patients with hsCRP levels of 4 mg/L or above exhibited an adjusted OR of 2.14 (95% CI: 1.63, 2.81) in comparison to patients with hsCRP levels below 4 mg/L, with a P-value of less than 0.001. Similarly, in all but the drinkers subgroup, there was a significant association between hsCRP levels and the risk of fibrosis (P < 0.05). Nevertheless, no significant interaction was observed in any subgroups except the alcohol subgroup (P for interaction = 0.012). In the analysis of cirrhosis (**Figure 4C**), patients with hsCRP levels ≥4 mg/L exhibited an adjusted OR of 1.95 (95% CI: 1.32, 2.90) in comparison to patients with hsCRP levels <4 mg/L, with a P-value of 0.001. Similarly, in the alcohol subgroup, the correlation between hsCRP levels and the risk of cirrhosis in non-drinkers was statistically significant (P < 0.001), with a notable between-group interaction (P for interaction = 0.030). No significant interaction was identified in the remaining subgroups (P for interaction > 0.05).

### 3.8 ROC curves of hsCRP in predicting hepatic fibrosis in MASLD patients

**Figure 5:**
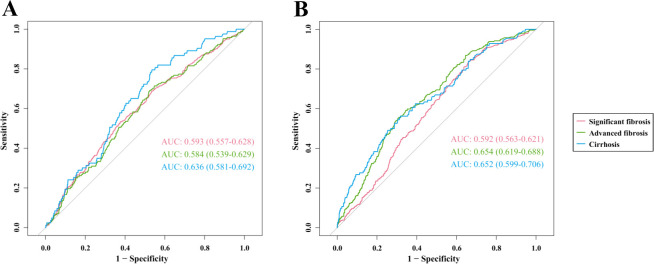
ROC curves of hsCRP for predicting hepatic fibrosis in patients with MASLD. **(A)** ROC curves for predicting significant fibrosis, advanced fibrosis, and cirrhosis in the US cohort; **(B)** ROC curves for predicting significant fibrosis, advanced fibrosis, and cirrhosis in the Chinese cohort. ROC, Receiver operating characteristics; hsCRP, high-sensitivity C-reactive protein; MASLD, Metabolic dysfunction-associated steatotic liver disease; AUC, Area under receiver operating characteristics curve.

In the US cohort (**Figure 5A**), the area under the curve (AUC) values of hsCRP for predicting significant fibrosis, advanced fibrosis, and cirrhosis were 0.593 (95% CI: 0.557-0.628), 0.584 (95% CI: 0.539-0.629), and 0.636 (95% CI: 0.581-0.692), respectively. These AUC values indicate that hsCRP has some degree of discriminatory power in predicting the degree of hepatic fibrosis in US patients with MASLD, particularly in the prediction of cirrhosis. In the Chinese cohort (**Figure 5B**), the AUC values of hsCRP for predicting significant fibrosis, advanced fibrosis, and cirrhosis were 0.592 (95% CI: 0.563-0.621), 0.654 (95% CI: 0.619-0.688), and 0.652 (95% CI: 0.599-0.706), respectively. As with the US cohort, these findings illustrate the effectiveness of hsCRP in forecasting the degree of hepatic fibrosis in Chinese patients with MASLD. However, the comparatively low AUC values suggest that its predictive accuracy is constrained.

## 4 Discussion

In this study, we comprehensively analyzed data from both the US and the Chinese cohorts to investigate the potential association between hsCRP and hepatic fibrosis in patients with MASLD. The study’s findings indicated a notable nonlinear correlation between hsCRP levels and the degree of hepatic fibrosis in patients with MASLD, as observed in both the US and Chinese cohorts. Additionally, the study identified specific inflection point values. Specifically, the risk of significant fibrosis, advanced fibrosis, and cirrhosis in patients with MASLD was significantly elevated with increasing hsCRP levels within a specific range, and this association remained significant after adjusting for multiple confounding variables. These findings further substantiate the utility of hsCRP as a potential predictor of hepatic fibrosis in patients with MASLD.

CRP, a pivotal pro-inflammatory factor, plays a pivotal role in various pathological conditions. It has been demonstrated that the pentameric form of CRP (pCRP) can dissociate into a pro-inflammatory monomeric form (mCRP) under specific shear or inflammatory conditions, thereby further activating the inflammatory response (23–25). For instance, in aortic stenosis (AS), shear force-induced dissociation of pCRP activates endothelial cells and platelets, leading to inflammation and thrombosis (24). Similarly, mCRP exacerbates localized tissue damage in atherosclerosis and myocardial infarction by binding to cell membrane phospholipids, inducing leukocyte adhesion and inflammatory cell activation (25). Additionally, mCRP interacts with lipid rafts in endothelial cell membranes to regulate cytokine release and endothelial dysfunction, further promoting inflammatory responses (26).

Some studies have indicated a positive correlation between elevated hsCRP levels, a sensitive marker of the inflammatory response, and the degree of hepatic fibrosis in patients with NAFLD (18, 20, 21). The present study not only corroborates previous findings but also underscores the significance of hsCRP in the context of MASLD. These findings further reinforce the notion that chronic low-grade inflammation plays a pivotal role in the progression of MASLD to hepatic fibrosis. Furthermore, the results of the present study align with a series of observational studies that have identified an association between hsCRP levels and cardiometabolic risk factors, including hypertension, diabetes, and dyslipidemia. These risk factors are also strongly associated with the progression of hepatic fibrosis in MASLD (15–17).

Some biological mechanisms may mediate the relationship between hsCRP and hepatic fibrosis in patients with MASLD. Firstly, elevated levels of hsCRP, a nonspecific inflammatory marker, typically indicate the presence of a low-grade inflammatory response within the body (13, 14). In patients with MASLD, persistent hepatic inflammation represents a pivotal factor in the progression of hepatic fibrosis (11). The infiltration and activation of inflammatory cells release a variety of pro-inflammatory cytokines and chemokines that activate hepatic stellate cells (HSCs), prompting their transformation into myofibroblasts and secretion of large quantities of extracellular matrix (ECM), which in turn drive the onset and progression of hepatic fibrosis (9, 11). Secondly, insulin resistance, a core feature of MASLD, is closely associated with elevated hsCRP levels (27). Insulin resistance may result in aberrant hepatic lipid and glucose metabolism, exacerbating hepatic inflammation and fibrosis (28). Additionally, insulin resistance may directly promote the activation and proliferation of HSCs through the activation of signaling pathways such as c-Jun N-terminal kinase and mechanistic target of rapamycin, thereby further accelerating the process of hepatic fibrosis (29–31). Moreover, patients with MASLD frequently present with dyslipidemia, which can result in the accumulation of lipids within the liver. The accumulation of lipids is susceptible to peroxidation in the presence of oxidative stress, generating many oxidation products and free radicals. These oxidation products and free radicals not only directly damage hepatocyte membranes, mitochondria, and other cellular organelles but also activate inflammatory signaling pathways, thus exacerbating hepatic inflammation and fibrosis (28, 32). hsCRP, as a component of the inflammatory response, and its elevated level may reflect the state of this oxidative stress and lipid peroxidation. Furthermore, alterations in the intestinal microbiota are strongly linked to the onset of MASLD and may impact hsCRP levels. Metabolites produced by the intestinal flora, including short-chain fatty acids and bile acids, have the potential to influence the metabolic and inflammatory state of the liver, which in turn affects hepatic fibrosis (33–35).

The present study also identified findings that differed from those observed in existing studies. Specifically, the application of RCS analysis identified a potential inflection point value for the dose-response relationship between hsCRP levels and hepatic fibrosis. This inflection point value may vary across geographic and ethnic populations. In the US cohort, the inflection point was approximately 9 mg/L, whereas in the Chinese cohort, it was 4 mg/L. This finding suggests that the effect of hsCRP on hepatic fibrosis may exhibit a threshold effect in different populations. That is to say; when the hsCRP level is lower than the inflection point, its elevation will significantly increase the risk of hepatic fibrosis. Conversely, when it exceeds the inflection point, its contributing effect on the risk of hepatic fibrosis may diminish or no longer be significant. These findings have important theoretical and practical implications for developing screening and monitoring strategies for hepatic fibrosis based on hsCRP levels.

Furthermore, this study investigated the impact of hsCRP levels on the likelihood of hepatic fibrosis in patients with varying characteristics of MASLD through subgroup analysis. The results demonstrated notable discrepancies in the correlation between hsCRP levels and the possibility of hepatic fibrosis across subgroups with disparate characteristics. For instance, the impact of hsCRP levels on the risk of hepatic fibrosis was more pronounced in the subgroups of non-smoking and non-drinking patients, and there was a significant between-group interaction. This phenomenon may be attributed to the fact that there were fewer potential confounding factors in participants who were non-smokers and non-drinkers compared to individuals who smoked or drank. This may have resulted in a clearer and stronger association between hsCRP levels and hepatic fibrosis risk. These findings offer novel insights into the personalized assessment of hepatic fibrosis risk in patients with MASLD, which can assist clinicians in formulating more precise and efficacious therapeutic regimens tailored to individual patient profiles.

Moreover, this study assessed the effectiveness of hsCRP in predicting the degree of hepatic fibrosis in patients with MASLD. Specifically, the AUC values of hs-CRP in predicting significant fibrosis, advanced fibrosis, and cirrhosis in patients with MASLD were all approximately 0.6. These findings indicate that hsCRP, when used as a single predictor, exhibits some discriminatory efficacy in differentiating between the various stages of hepatic fibrosis in patients with MASLD. However, its overall predictive accuracy remains limited. In light of these findings, we propose that hsCRP should be employed in conjunction with other predictors of hepatic fibrosis in clinical practice. Furthermore, we recommend that the accuracy and specificity of prediction be enhanced by comprehensively considering a range of biomarkers.

A substantial body of research has demonstrated the considerable therapeutic potential of CRP in the treatment of inflammation-related diseases. The suppression of the pro-inflammatory effects of CRP through diverse mechanisms has been identified as a highly effective anti-inflammatory strategy (23). For instance, the development of specific small-molecule inhibitors (e.g., 1,6-bis(phosphocholine)-hexane) has been shown to impede CRP function and mitigate tissue damage in myocardial infarction and stroke (36). In addition, selective CRP-scavenging therapies, which expeditiously reduce circulating CRP levels through blood purification techniques, have significantly improved clinical symptoms and reduced mortality among patients with severe pneumonia caused by COVID-19 (37–39). Furthermore, low molecular weight CRP inhibitors offer novel insights for anti-inflammatory therapy by mimicking the structure of phospholipids, specifically binding to the phosphorylcholine-binding pocket of CRP and inhibiting its conversion to pro-inflammatory isoforms (40). These studies validate the feasibility of CRP as a therapeutic target and demonstrate its promising broad application in various inflammatory diseases, providing strong support for the development of novel anti-inflammatory therapies.

While the findings of this study are consistent across two large cohorts, it is important to acknowledge the limitations of the study design. First, due to the cross-sectional study design, it was impossible to establish a definitive causal relationship between hsCRP and hepatic fibrosis. Accordingly, future studies must adopt a longitudinal design to validate this association further. Secondly, although the study included two geographically diverse cohorts, the sample size was still insufficient. It may have been affected by selection bias and unmeasured confounders, which may have impacted the study results. Furthermore, there were discrepancies in hsCRP measurements between the two cohorts, which may have negatively impacted the direct comparison of results. It is recommended that future studies employ a prospective cohort design to not only validate the relationship between hsCRP and hepatic fibrosis in patients with MASLD in greater depth but also to explore the applicability of hsCRP in populations of different races and regions. Ultimately, as this study concentrated on hsCRP as a singular inflammatory marker, future research could be expanded to investigate the correlation between other inflammatory markers and hepatic fibrosis and the potential utility of combining multiple inflammatory markers for hepatic fibrosis risk assessment.

## 5 Conclusion

In conclusion, the present study employed cross-regional and large-scale data analysis to investigate the correlation between hsCRP levels and hepatic fibrosis in MASLD patients. The findings revealed a potential dose-response relationship and variability in different subgroups. These findings underscore the pivotal role of inflammation in the progression of MASLD and offer a novel perspective and empirical basis for risk assessment of hepatic fibrosis in patients with MASLD. They also provide a valuable reference for future research directions. Further research is required to elucidate the mechanisms by which hsCRP may contribute to hepatic fibrosis. Additionally, more extensive longitudinal studies encompassing diverse geographical and ethnic groups must confirm these findings. This study aims to validate the findings and elucidate the underlying pathophysiological mechanisms, providing more effective preventive and therapeutic strategies for MASLD patients.

## Data Availability

The US cohort data presented in this study can be found in online repositories. The names of the repository/repositories and accession number(s) can be found below: https://www.cdc.gov/nchs/nhanes/index.htm. The Chinese cohort data utilized and analyzed in the present study are accessible from the corresponding author/s without undue reservation.
